# Transcriptional regulation of miR-15b by c-Rel and CREB in Japanese encephalitis virus infection

**DOI:** 10.1038/srep22581

**Published:** 2016-03-02

**Authors:** Bibo Zhu, Jing Ye, Usama Ashraf, Yunchuan Li, Huanchun Chen, Yunfeng Song, Shengbo Cao

**Affiliations:** 1 State Key Laboratory of Agricultural Microbiology, Huazhong Agricultural University, Wuhan, Hubei, 430070, P.R China; 2 Laboratory of Animal Virology, College of Veterinary Medicine, Huazhong Agricultural University, Wuhan, Hubei, 430070, P.R China; 3 The Cooperative Innovation Center for Sustainable Pig Production, Huazhong Agricultural University, Wuhan, Hubei, 430070, P. R. China

## Abstract

MicroRNAs (miRNAs) have been well known to play diverse roles in viral infection at the level of posttranscriptional repression. However, much less is understood about the mechanism by which miRNAs are regulated during viral infection. It is likely that both host and virus contain factors to modulate miRNA expression. Here we report the up-regulation of microRNA-15b (miR-15b) *in vitro* upon infection with Japanese encephalitis virus (JEV). Analysis of miR-15b precursor, pri-miR-15b and pre-miR-15b, suggest that the regulation occurs transcriptionally. Further, we identified the transcriptional regulatory region of miR-15b that contains consensus binding motif for NF-κB subunit c-Rel and cAMP-response element binding protein (CREB), which are known as transcription factor to regulate gene expression. By promoter fusion and mutational analyses, we demonstrated that c-Rel and CREB bind directly to the promoter elements of miR-15b, which are responsible for miR-15b transcription in response to JEV infection. Finally, we showed that pharmacological inhibition of ERK and NF-κB signaling pathway blocked induction of miR-15b in JEV infection, suggesting important roles of ERK and NF-κB pathway in the regulation of miR-15b gene. Therefore, our observations indicate that induced expression of miR-15b is modulated by c-Rel and CREB in response to JEV infection.

MicroRNAs (miRNAs) are approximately 22 nucleotides (nt) evolutionarily conserved non-coding small RNAs^1^. Similar to other RNA molecules, most of miRNAs are initially transcribed by RNA polymerase II^2^. The primary transcripts of miRNAs (pri-miRNAs) are cleaved by RNAase III Drosha in the nucleus to liberate ~70 nt precursors (pre-miRNAs). The pre-miRNAs are then shuttled into the cytoplasm, and processed by the RNAase III Dicer into mature miRNAs. The mature miRNAs are loaded into an Argonature protein to form the RNA-induced silencing complex (RISC), where they guide the recognition through base pairing and translational repression and/or degradation of target mRNAs^1^,^3^. In mammals, miRNAs have been associated with a wide range of biological processes, such as cell differentiation, cancer, proliferation, and apoptosis^4^,^5^,^6^. In addition to their regulatory roles in diverse biological pathways, miRNAs have also been implicated with viral infections^7^. For example, miR-26a induces a significant inhibition of Porcine Reproductive and Respiratory Syndrome virus replication by upregulating type I interferon signaling pathway^8^. mir-23b promotes Avian Leukosis virus replication through targeting IRF1^9^. miR-122 stimulates Hepatitis C virus RNA synthesis by rebalancing RNA engagement in RNA versus protein synthesis^10^. Although the function of miRNAs has been widely studied during viral infection, the knowledge about how miRNA genes themselves are regulated has comparatively lagged behind.

The majority of miRNA genes are located in intergenic regions or in antisense orientation to annotated genes, indicating that they form independent transcription units^2^. Most of other miRNAs are found in intronic regions, which may be transcribed as part of the host genes^11^. Expression of intronic miRNAs largely coincides with the transcription of their host genes and they may be coregulated and generated from a common precursor transcript^12^. Accumulating evidence has shown that the expression of miRNAs is often subject to regulation by transcriptional factors and co-regulators. For example, NF-κB p65 subunit binds to the promoter element of a subset of miRNA genes and transcriptionally regulates their expression in response to LPS stimulation^13^,^14^. A proto-oncogenic transcription factor CREB directly binds to the regulatory sequences of miR-23a and enhance the expression of miR-23a^15^. Moreover, miR-100, -146a and -150 are reported to be novel p53 and NF-κB p65/RelA responsive miRNAs^16^. Other transcription factors, such as c-Myc, E2F, STAT3, and C/EBPa, are also found to modulate the expression of miRNAs^17^,^18^,^19^,^20^,^21^.

Japanese encephalitis virus (JEV), a member of the family *Flaviviridae*, is an enveloped, single-stranded, and positive-sense RNA virus^22^. The genomic RNA with approximately 11 kb in length encodes a single polyprotein that is processed into three structural proteins-the capsid (C), membrane (M) and envelop (E) proteins, and seven nonstructural proteins-NS1, NS2A, NS2B, NS3, NS4A, NS4B, and NS5^22^. JEV is the most prevalent cause of viral encephalitis in the Asia-Pacific region, with 30,000–50,000 cases (mostly children) and a high fatality rate of 30% being reported annually^23^. Japanese Encephalitis is characterized by profound neuronal damage along with intense microgliosis and astrogliosis. Neurological complications such as inflammation and neuronal death contribute to the mortality and morbidity^24^,^25^. Recently, a number of studies have been focusing on the role of miRNAs in JEV pathogenesis. miR-29b, -155, -146a and -15b were observed to be up-regulated following JEV infection, and they can modulate JEV-triggered neuroinflammation by targeting genes involved in inflammatory signal pathway^26^,^27^,^28^,^29^. However, the mechanisms by which these miRNAs are regulated after JEV infection are poorly understood.

Our previous study highlighted the property of miR-15b as a pro-inflammatory miRNA, with a critical role in regulating JEV-inflammatory response in glial cells and viral challenged mouse model^29^. To dissect the effects of miR-15b upon JEV infection, it would be crucial to understand how miR-15b gene is regulated. In this report, we examined the potential mechanism in regulation of miR-15b expression. The data demonstrated that the precursor miRNAs, pri-miR-15b and pre-miR-15b, as well as mature miR-15b were up-regulated, suggesting that regulation of miR-15b occurs transcriptionally. Promoter analysis of miR-15b gene revealed that c-Rel and CREB play important roles in induction of miR-15b transcription in JEV infection. In addition, we have determined that the induction of miR-15b depends on RIG-I signaling, and downstream ERK and NF-κB pathways are involved in regulation of miR-15b. The present study is the first to elucidate the transcriptional regulation of miR-15b by JEV infection, and that would provide clues to the further understanding of complex functions operated by miR-15b.

## Results

### miR-15b is induced in JEV-infected HeLa cells

**Figure 1:**
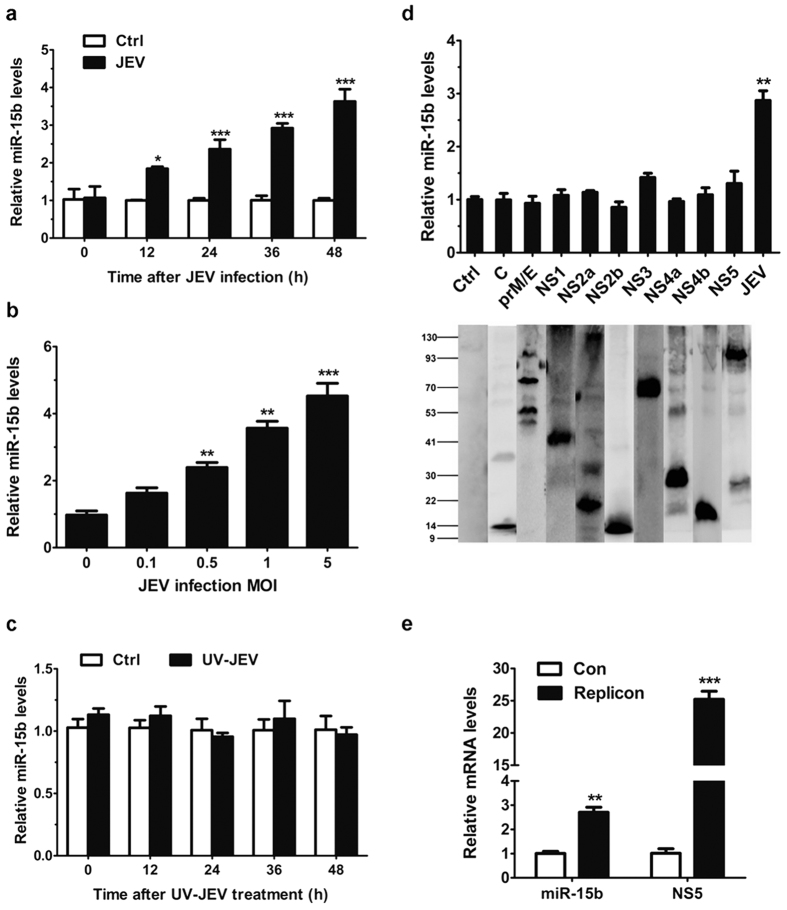
miR-15b is upregulated in JEV-infected HeLa cells. (**a,b**) HeLa cells were infected with JEV at an MOI of 1 for the indicated times (**a**), or at indicated MOIs for 36 h (**b**). qRT-PCR was performed to determine the expression of miR-15b. The amount of miR-15b was obtained by normalizing to U6 levels in the samples. Data are expressed as the amount of miR-15b in JEV-infected samples relative to the control non-infected samples. (**c**) HeLa cells were incubated with UV-irradiated inactive JEV for the indicated times. The expression of miR-15b was detected by qRT-PCR. (**d**) HeLa cells were transfected with the plasmids encoding each of the 9 JEV proteins as indicated for 36 h. The levels of miR-15b were measured by qRT-PCR (upper panel). The protein expression patterns of plasmids expressing individual JEV proteins were detected by Western blot (lower panel). The vector transfected cells were used as controls and values from these cells were regarded as 1. JEV-infected cells were used as positive control. (**e**) HeLa cells were transfected with JEV replicon RNA for 36 h. Total cellular RNA was extracted and subjected to qRT-PCR. The levels of miR-15b and JEV RNA (NS5) were determined. All the data represent means ± SD from triplicate wells. Statistical analysis for (**a**,**e**) was carried out by 2-way ANOVA with subsequent *t* tests using a Bonferroni post-tests. Statistical analysis for (**b**,**d**) was carried out by a Student *t* test. *p < 0.05, **p < 0.01, ***p < 0.001.

Several studies have demonstrated changes in host miRNA expression in response to JEV infection^26^,^27^,^28^. We previously described the altered expression of miR-15b in glial cells and mouse brain following JEV infection^29^. To delineate the kinetics of miR-15b induction in HeLa cells, mature miR-15b were assayed over a 48-h time course after JEV infection. As shown in Fig. 1a, mature miR-15b was expressed constitutively in the cells, and the levels of miR-15b increased significantly in a time-dependent manner after JEV stimulation. Similar to our previous study, miR-15b was also up-regulated in a dose-dependent manner (Fig. 1b). Moreover, cells inoculated with UV-irradiated inactivated JEV were used for miR-15b detection and the expression level of miR-15b was shown to have no significant changes (Fig. 1c). Since JEV infection is involved in the regulation of miR-15b expression. We next wanted to determine which, if any, of the JEV proteins was responsible for such regulation. HeLa cells were transfected with plasmids expressing each of the JEV proteins. qRT-PCR analysis showed that exogenous expression of JEV proteins can not alter the levels of miR-15b (Fig. 1d). To further determine the effect of viral RNA on miR-15b induction, a transient replicon system of JEV was used. HeLa cells were transfected with the JEV subgenomic replicon and the miR-15b expression was detected. The results showed that the replicon RNA increased the levels of miR-15b compared with that in control cells (Fig. 1e). Taken together, these results indicated that up-regulation of miR-15b in JEV infected HeLa cells may rely on viral replication. To explore the biological significance of miR-15b upregulation in HeLa cells in response to JEV infection, the roles of miR-15b played in the production of JEV-induced inflammatory cytokines and viral replication were investigated. Consistent with our previous study^29^, miR-15b positively regulates the expression of JEV-triggered inflammatory cytokines, and that manipulation of miR-15b in cells has no significant effect on JEV replication (see Supplementary Fig. S1).

### Differential expression of primary transcripts of JEV-induced miR-15b

**Figure 2:**
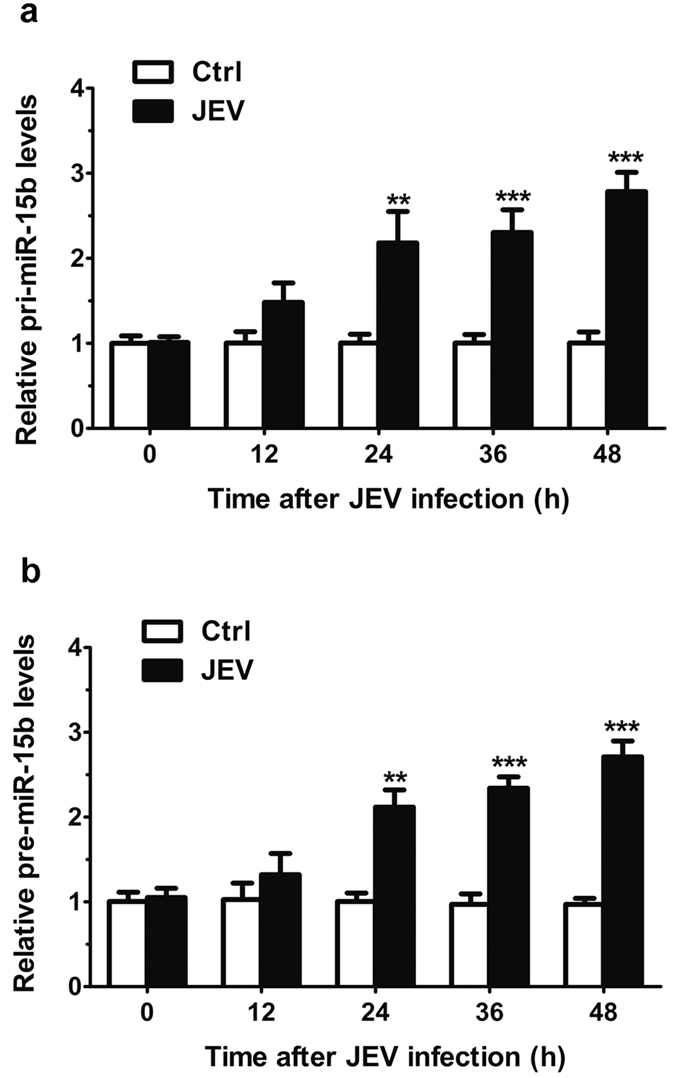
Altered expression of primary transcripts of miR-15b in HeLa cells with JEV infection. (**a,b**) HeLa cells were infected with JEV at an MOI of 1 for the indicated lengths of time. qRT-PCR was performed to detect the expression of pri-miR-15b (**a**) and pre-miR-15b (**b**). The amount of pri-miR-15b and pre-miR-15b was obtained by normalizing to the level of β-actin in the samples. The non-treated cells were used as controls and values from these cells were regarded as 1. Values are expressed as mean ± SD from triplicate wells. Statistical analysis was carried out by 2-way ANOVA with subsequent *t* tests using a Bonferroni post-tests. **p < 0.01; ***p < 0.001.

Given the induction of miR-15b in response to JEV infection, we further measured the expression of primary miR-15b transcript (pri-miR-15b) and miR-15b precursor (pre-miR-15b), from which the mature miR-15b is processed, in JEV-infected HeLa cells. We analyzed the kinetics of alterations in the pri-miR-15b and pre-miR-15b. Expression of pri-miR-15b showed a time-dependent increase in cells following JEV infection (Fig. 2a). Similarly, the levels of pre-miR-15b were also up-regulated after JEV infection (Fig. 2b). Thus, the induction of pri-miR-15b and pre-miR-15b coincided with that of mature miR-15b, suggesting that the upregulation of miR-15b may occur at the transcriptional level.

### Identification of the promoter region for miR-15b

**Figure 3:**
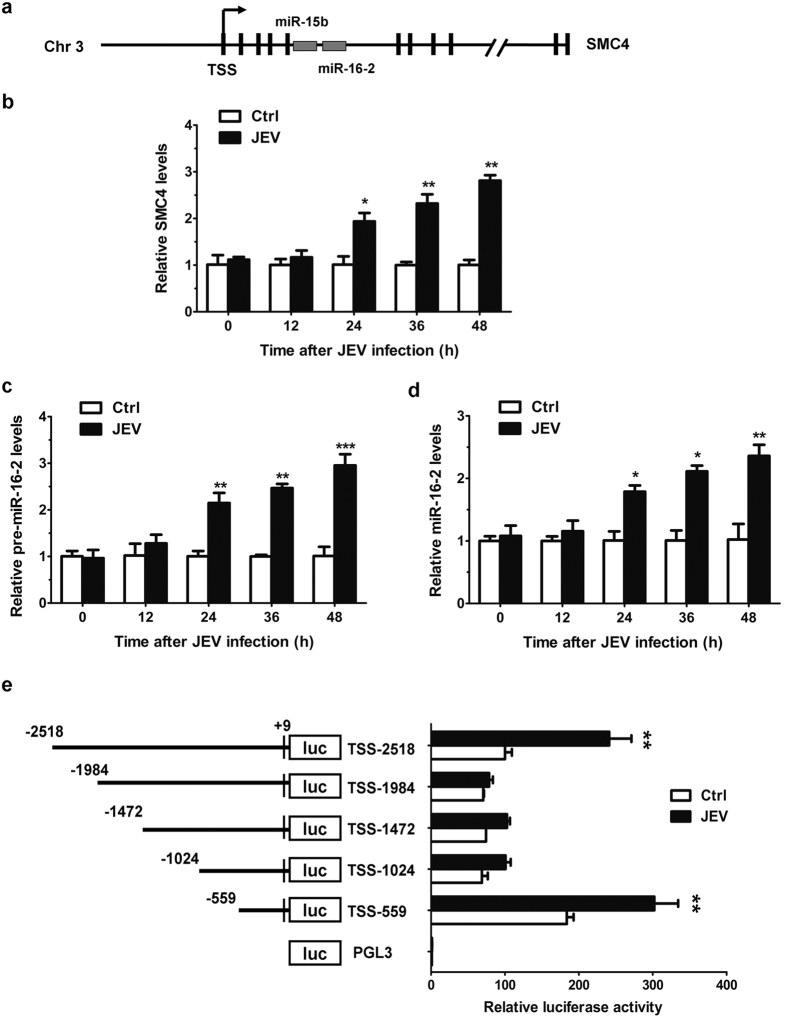
Identification of the boundary of miR-15b promoter region. (**a**) The structure of the human DNA loci expressing miR-15b is shown. miR-15b and miR-16-2 cluster resides on chromosome 3 in intron 5 of host SMC4 gene. TSS, transcriptional start site. (**b–d**) HeLa cells were infected with JEV at an MOI of 1 for the indicated times. qRT-PCR was performed to measure the expression of SMC4 (**b**), pre-miR-16-2 (**c**) and miR-16-2 (**d**). The non-infected cells were used as controls and values from these cells were regarded as 1. (**e**) Hela cells were transfected with miR-15b promoter reporters containing various lengths of the miR-15b promoter region for 24 h and then either left uninfected or infected with JEV at an MOI of 1. 24 h later, luciferase activity was measured, and results were expressed as percentages of TSS-2518 basal activity (regarded as 100%). Similar results were obtained in three independent experiments. Statistical analysis was carried out by 2-way ANOVA with subsequent *t* tests using a Bonferroni post-tests. *p < 0.05, **p < 0.01, ***p < 0.001.

As JEV infection regulates the expression of pri-miR-15b, the underlying mechanism which is responsible for such regulation was determined. The genomic information and location of miR-15b were obtained from miRBase^30^. miR-15b is clustered miRNA and have cluster partners miR-16-2. miR-15b and miR-16-2 cluster resides on chromosome 3 in intron 5 of host SMC4 gene^21^, which encodes for a protein that functions mainly in M phase and is essential for mitosis specific chromatin condensation^31^. The expression of miR-15b-16-2 cluster is usually co-regulated with SMC4 gene^21^,^32^. A schematic representation of miR-15b location is shown in Fig. 3a. Since miR-15b is co-localized with miR-16-2 in the intron of SMC4 gene, the expression of SMC4, pre-miR-16-2 and miR-16-2 were examined in response to JEV infection by qRT-PCR. JEV infection resulted in a time-dependent increase in SMC4 mRNA expression (Fig. 3b). Expression of pre-miR-16-2 and miR-16-2 were also up-regulated after JEV infection (Fig. 3c,d). To evaluate the transcriptional regulation of miR-15b, we then sought to analyze the promoter region of miR-15b. To define the boundaries of the minimal promoter region, an approximately 2.5-kb DNA fragment containing non-coding sequences upstream from the SMC4 transcriptional initiation site (TSS) was selected for promoter mapping. Luciferase reporter genes with different lengths of the miR-15b putative promoter regions were constructed and transfected into HeLa cells to determine the basal and JEV-induced promoter activities. As shown in Fig. 3e, full-length reporter (TSS-2518) displayed both basal promoter activity and clear JEV inducibility, which was decreased by three truncations of this full-length region, TSS-1984, TSS-1472 and TSS-1024. However, the reporter TSS-559 had a higher luciferase activity in both mock-infected HeLa cells and JEV-infected HeLa cells, comparable to that of the longest reporter TSS-2518, suggesting that TSS-559 possesses fully intact promoter activity. Of note, TSS-559 reporter spanning the −559 to + 9 bp region (where +1 corresponds to SMC4 TSS) was found to be miR-15b promoter that was responsive to JEV infection, and chosen for further analysis.

### C-Rel and CREB elements regulate miR-15b promoter activity

**Figure 4:**
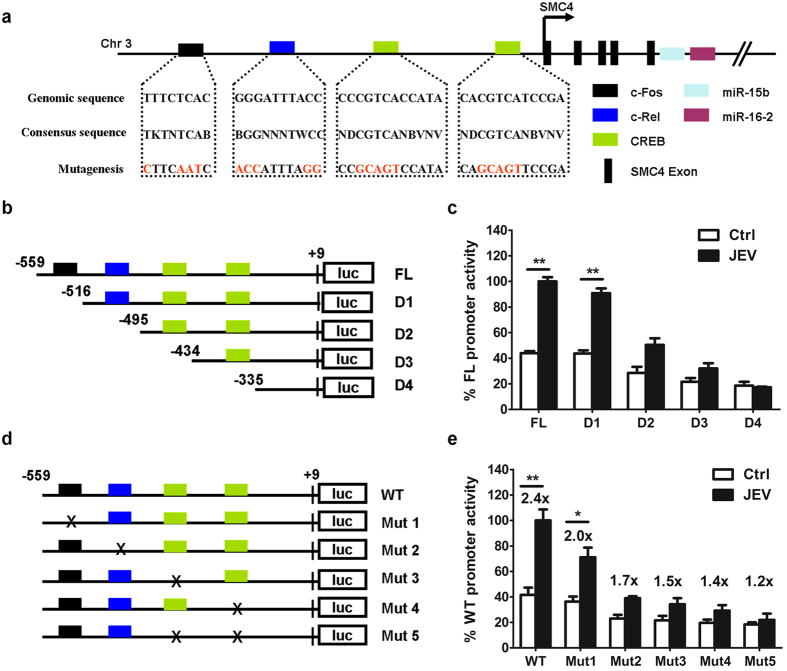
C-Rel and CREB regulate miR-15b promoter activity. (**a**) Schematic diagram of miR-15b genomic loci on human chromosomes 3. Putative binding sites of c-Fos, c-Rel, and CREB transcriptional factors (TFs) are shown as boxes. The sequences of mutated sites are shown under the locus diagram. (**b**) Schematic representation of deletion mutants D1 to D4 in the full-length miR-15b promoter (FL). (**c**) The promoter reporter constructs (100 ng) described in (**b**) together with PRL-TK (10 ng) were co-transfected in Hela cells. At 24 h post-transfection, cells were infected with medium or JEV for another 24 h. Samples were collected and analyzed for dual luciferase activity. Results were plotted as firefly luciferase activity (standardized to Renilla luciferase activity) and expressed as percentages of JEV-inducible FL promoter activity (100%). (**d**) Schematic representation of point mutations (Mut1 to Mut 5) in the wild-type promoter (WT). Mutations, disrupting transcription factor binding, introduced in the promoter construct, which was indicated by x. (**e**) Hela cells were co-transfected with mutations in (**d**) and PRL-TK as described in (**c**). Fold increase after JEV infection relative to basal condition was indicated for each construct. Error bars represent the standard deviation (SD) calculated from results of at least three independent experiments. Statistical analysis was carried out by 2-way ANOVA with subsequent *t* tests using a Bonferroni post-tests. *p < 0.05; **p < 0.01.

Bioinformatics analyses of the miR-15b promoter region (TSS-599) using MethPrimer^33^ and CONSITE^34^ software predicted several potential transcription factor-binding sites (TFBS). The putative binding sequences for three potential transcription factors, including c-Fos, c-Rel and CREB, were found within miR-15b promoter. The consensus binding sites and mutations were shown in Fig. 4a. To determine if the predicted transcription factors (TFs) were responsive to miR-15b transcription, a series of truncated promoter constructs including full-length (FL), deletion 1 (D1), D2, D3 and D4 were generated (Fig. 4b). In comparison with the full length promoter construct, deletion of the distal c-Fos binding site (D1) had no significant effect on constitutive or inducible luciferase activity, suggesting that it was not necessary for miR-15b promoter activation. However, the promoter constructs, including deletion of c-Rel and CREB binding site (D2, D3 and D4), exhibited significantly less response to JEV treatment (Fig. 4c). To further confirm these findings, a panel of five different constructs (with specific point mutations) including mutation 1 (Mut1), Mut2, Mut3, Mut4, and Mut5 were generated in the context of the FL promoter to assess the functional role of the specific predicted elements (Fig. 4d). As shown in Fig. 4e, mutation disturbing the c-Fos element binding site (Mut1) did not affect basal activity but slightly reduce JEV-induced promoter activity, whereas mutations affecting c-Rel (Mut2) or CREB binding sites (Mut3 and Mut4) abrogated JEV-mediated induction of miR-15b promoter activity. Furthermore, activation of the miR-15b promoter by JEV was also impaired by mutations in both CREB binding sites (Mut5) (Fig. 4e). These data suggest that the activation of miR-15b promoter by JEV infection requires both c-Rel and CREB element.

**Figure 5:**
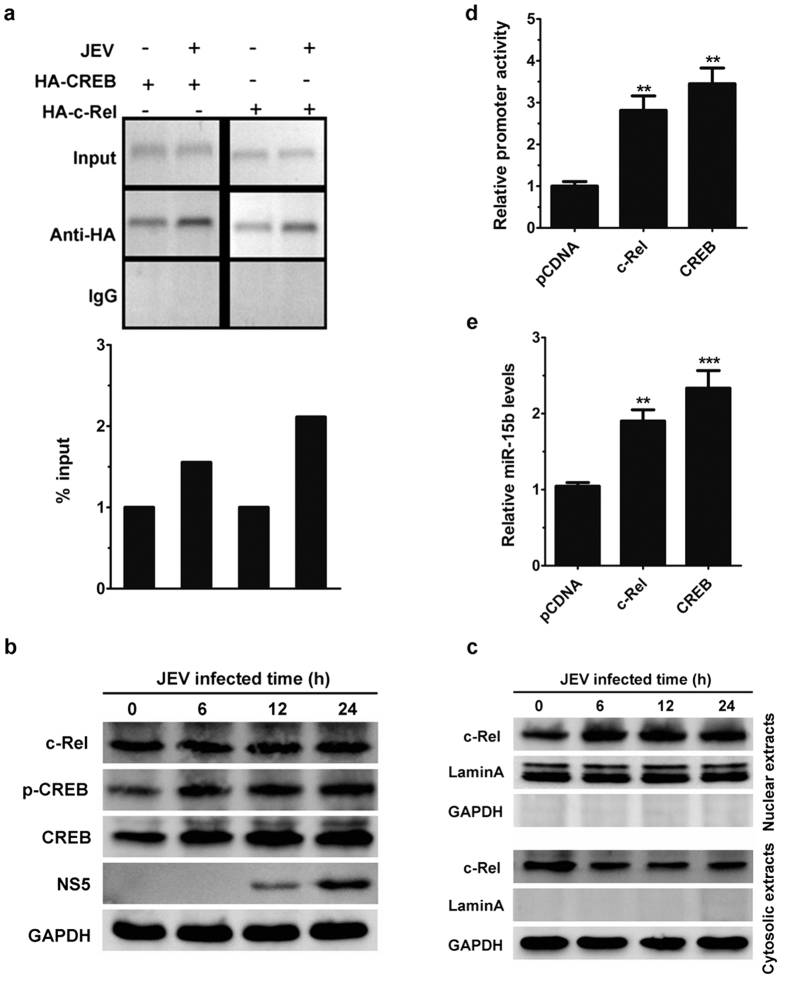
C-Rel and CREB directly bind to miR-15b promoter *in vivo*. (**a**) Hela cells were transfected with plasmids encoding HA-tagged c-Rel or CREB for 24 h, and then infected with JEV. Fixed chromatin from HeLa cells were prepared and immunoprecipitated by anti-HA antibody or normal rabbit IgG. ChIP primers were designed to amplify the region containing both c-Rel and CREB binding site in the miR-15b promoter. PCR products were separated by acrylamide gel electrophoresis. Representative gels are shown in the upper panel and densitometry analysis in the lower panel. (**b,c**) HeLa cells were infected with JEV for the indicated times. The expression of c-Rel, phosphorylated CREB, total CREB, NS5 and GAPDH in the whole cell lysates were monitored by immunoblotting (**b**). The nuclear extracts and cytosolic extracts were isolated and subjected to immunoblotting with antibodies against c-Rel, GAPDH (as a cytoplasmic marker), and laminA (as a nuclear marker) (**c)**. **(d**) HeLa cells were co-transfected with miR-15b promoter reporter plasmid, PRL-TK vector and c-Rel and CREB expressing plasmid for 24 h. The miR-15b promoter activities were measured by dual luciferase assays. (**e**) The expression of miR-15b was determined by qRT-PCR in HeLa cells overexpressing c-Rel or CREB. Results were represented as fold change over empty vector control. Error bars represent the standard deviation (SD) calculated from results of at least three independent experiments. Statistical analysis was carried out by a Student *t* test. **p < 0.01; ***p < 0.001.

To further substantiate the role that c-Rel and CREB may play in JEV-induced activation of miR-15b promoter, a chromatin immunoprecipitation (ChIP) assay was performed. To this end, isolated chromatin from c-Rel and CREB transfected HeLa cells were immunoprecipitated, followed by PCR analysis. It was observed that c-Rel and CREB binds to the potential binding sites in non-infected HeLa cells (Fig. 5a). Moreover, JEV-treated cells showed an increase in the binding of c-Rel and CREB to the binding sites in miR-15b promoter (Fig. 5a). In contrast, immunoprecipitation of chromatin samples with control rabbit IgG did not show any amplification (Fig. 5a). To further confirm the binding of c-Rel and CREB to the miR-15b promoter, ChIP assays were performed in HeLa cells without c-Rel and CREB transfection. For this analysis, c-Rel and CREB antibodies or control rabbit IgG were used. Consistently, both c-Rel and CREB bound to the miR-15b promoter, and this activity was increased in cells with JEV infection (see Supplementary Fig. S2). Furthermore, the expression levels of c-Rel and CREB were determined in JEV-infected HeLa cells. The level of CREB, but not c-Rel, was increased by JEV infection (Fig. 5b). The activation of c-Rel and CREB was analyzed in cells with JEV infection. The results showed that JEV infection enhanced CREB phosphorylation (Fig. 5b). It is noteworthy that the translocation of c-Rel from the cytoplasm to the nucleus was found to be increased in JEV infection (Fig. 5c). In addition, overexpression of c-Rel or CREB enhanced the transcriptional activity of miR-15b promoter as well as the expression of the endogenous miR-15b (Fig. 5d,e). Taken together, these data suggest that c-Rel and CREB can physically bind to the promoter element of miR-15b and are important transcriptional regulators of miR-15b.

### Involvement of MAPK/ERK and NF-κB signaling pathways in JEV-induced miR-15b expression

**Figure 6:**
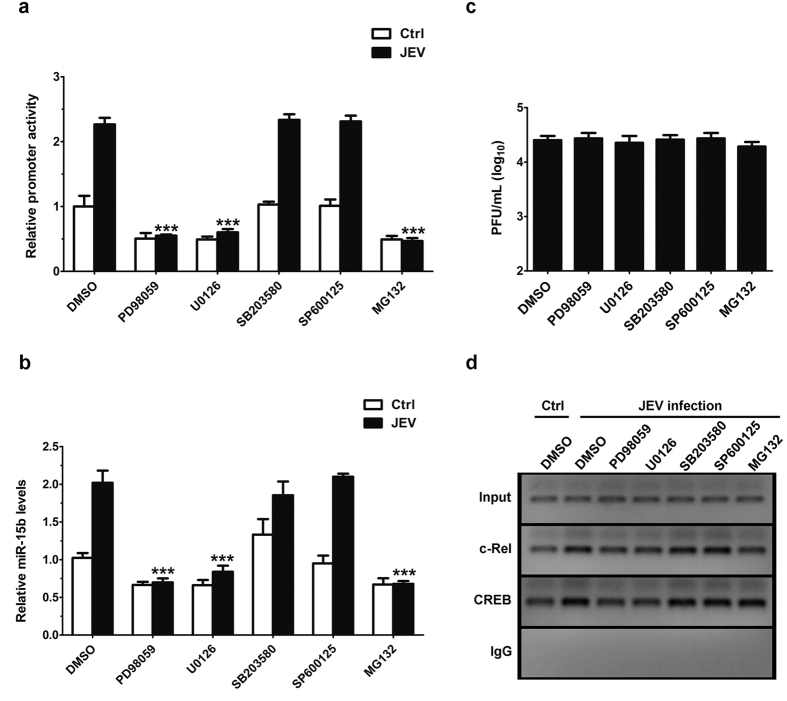
Analysis of signaling pathways involved in the regulation of miR-15b expression upon JEV infection. (**a**) Hela cells were transfected with miR-15b promoter for 24 h, and then either left uninfected or infected with JEV in the presence of signaling pathway specific inhibitors (10 μM each) as indicated. After 24 h, miR-15b promoter activity was measured by dual luciferase assays. (**b**) Hela cells were infected with JEV or mock-infected in the presence of signaling pathway specific inhibitors as indicated. The levels of miR-15b expression were detected by qRT-PCR All results are represented as fold change of the mock-infected group treated with DMSO, which was considered as 1. Error bars represent the standard deviation (SD) calculated from results of at least three independent experiments. Statistical analysis for (**a,b**) was carried out by 2-way ANOVA with subsequent *t* tests using a Bonferroni post-tests. ***p < 0.001. (**c**) Hela cells were treated as in (**b**). At 24 h post infection, the supernatants were collected and subject to plaque assay for the determination of viral titers. (**d**) ChIP analysis of JEV-infected HeLa cells in the presence of signaling pathway specific inhibitors as indicated, for assay of c-Rel and CREB binding to the miR-15b promoter. C-Rel antibody, CREB antibody or control rabbit IgG were used for immunoprecipitation of chromatin. The upper panel represents the amplification of input DNA before immunoprecipitation.

The role of MAPK and NF-κB signaling pathways is well known for the regulation of numerous important biological processes including the biogenesis of miRNAs at the cellular level^14^,^35^. To analyze whether these signaling pathways play an important role in the regulation of miR-15b expression induced by JEV, specific inhibitors of signaling pathways were employed. The results from luciferase activity assays showed that miR-15b promoter activity induced by JEV decreased significantly in cells treated with PD98059 (MEK-specific inhibitor), U0126 (ERK-specific inhibitor), or MG132 (NF-κB-specific inhibitor) with respect to DMSO treated cells (Fig. 6a). In contrast, cells treated with SB203580 (p38 MAPK -specific inhibitor) or SP600125 (JNK-specific inhibitor) had no appreciable inhibitory effect on miR-15b promoter activity induced by JEV (Fig. 6a). In order to confirm the results, qRT-PCR analyses were performed. The data also revealed significantly blockaded induction of miR-15b by JEV in the presence of PD98059, U0126, or MG132 (Fig. 6b). To exclude the possibility that the effects with inhibitors of signal pathways might be because of suppressed viral replication, we measured viral loads in JEV-infected HeLa cells in the presence of inhibitors. We observed non-significant changes in viral load in cells treated with inhibitors (Fig. 6c). To further investigate whether those inhibitors have any effects on the binding of c-Rel and CREB to the miR-15b promoter, ChIP assays were performed in HeLa cells treated with inhibitors. ChIP analysis showed that PD98059, U0126, and MG132 treatment decreased c-Rel binding to miR-15b promoter during JEV infection compared to DMSO treatment, but the binding activity was not significantly modulated by SB203580 or SP600125 treatment (Fig. 6d). Furthermore, the binding of CREB to the miR-15b promoter were suppressed by PD98059 and U0126, whereas the binding activity did not alter in SB203580, SP600125 or MG132 treated cells (Fig. 6d). As shown, amplification of the input DNA prior to immunoprecipitation was equivalent in all samples and the absence of signal in the control IgG immunoprecipitate confirmed the specificity of the anti-c-Rel and anti-CREB immunoprecipitation (Fig. 6d). These observations link the ERK and NF-κB signaling to the c-Rel- and CREB- mediated activation of miR-15b expression in response to JEV infection. Taken together, the results from these experiments indicate that ERK and NF-κB signaling pathways play important roles in the transcriptional regulation of JEV-triggered miR-15b.

### JEV infection up-regulates miR-15b expression through RIG-I-dependent pathway

**Figure 7:**
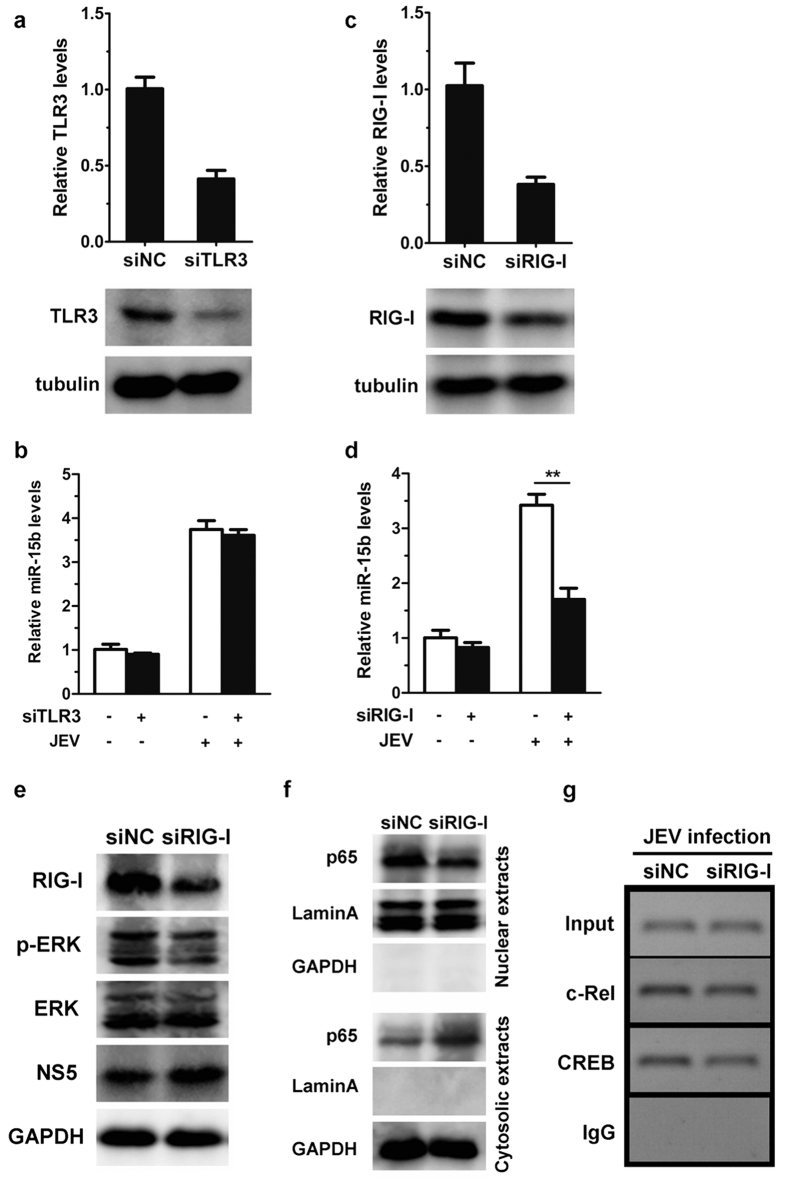
Induction of miR-15b by JEV depends on RIG-I. (**a,b**) Hela cells were transfected with either control siRNA (siNC) or siRNA targets TLR3 (siTLR3) for 24 h and then infected with JEV at an MOI of 1 for 48 h. RNA was extracted, and TLR3 transcript levels were determined by qRT-PCR and normalized to β-actin (**a**, upper panel). Cell lysates were extracted and immunoblotted for TLR3 and β-tubulin (**a**, lower panel). (**b**) The levels of miR-15b were detected by qRT-PCR and normalized to U6 levels. (**c,d**) HeLa cells were transfected with control siRNA (siNC) or RIG-I siRNA (siRIG-I) for 24 h, followed by 48 h of infection with JEV. RIG-I mRNA expression was assessed by qRT-PCR (**c**, upper panel) and RIG-I protein was detected by western blotting (c, lower panel). (**d**) The levels of miR-15b were measured by qRT-PCR. (**e,f**) HeLa cells were transfected with control siRNA (siNC) or RIG-I siRNA (siRIG-I) for 24 h and then infected with JEV at an MOI of 1 for 48 h. The expression of RIG-I, phosphorylated ERK, total ERK, NS5, and GAPDH in the whole cell lysates were monitored by immunoblotting (**e**). The nuclear extracts and cytosolic extracts were isolated and subjected to immunoblotting with antibodies against NF-κB p65, GAPDH (as a cytoplasmic marker), and laminA (as a nuclear marker) (**f**). (**g**) Hela cells were treated as in (**e**). ChIP assays were performed on cells extracts using antibodies against c-Rel and CREB or normal rabbit IgG followed by PCR amplification with primers against to the region containing both c-Rel and CREB binding site in the miR-15b promoter. PCR products were separated by acrylamide gel electrophoresis. All experiments were repeated at least three times with similar results. Statistical analysis was carried out by 2-way ANOVA with subsequent *t* tests using a Bonferroni post-tests. **p < 0.01.

Toll-like receptor 3 (TLR3) and retinoic acid inducible gene I (RIG-I) have been shown to recognize JEV infection, leading to activation of MAPK/ERK and NF-κB signaling events^36^,^37^. Furthermore, TLR3 and RIG-I are known to induce many miRNAs expression^38^,^39^. Thus, we speculate that increased expression of miR-15b following JEV infection may be mediated via TLR3 and/or RIG-I pathway. To confirm this hypothesis, we then assessed the contribution of the TLR3 pathway and the RIG-I pathway to the regulation of miR-15b expression. siRNAs were used to knockdown endogenous TLR3 and RIG-I expression, and the efficiency of TLR3 and RIG-I knockdown was evaluated by qRT-PCR and western blot analysis. As shown in Fig. 7a, a marked reduction in expression levels of TLR3 was observed with siRNA treatment, but JEV-induced miR-15b upregulation was intact in TLR3 knockdown cells (Fig. 7b). In contrast, induction of miR-15b by JEV challenge was markedly impaired in HeLa cells with reduced RIG-I, compared to cells transfected with non-targeting control siRNA (Fig. 7c,d). In addition, treatment with RIG-I-specific siRNA led to an increase in JEV load (see Supplementary Fig. S3), suggesting that the induction of miR-15b by JEV may through RIG-I.

To better understand the involvement of RIG-I in the induction of miR-15b, the activation of downstream signal proteins, ERK and NF-κB, were determined during JEV infection. As shown in Fig. 7e,f, following RIG-I knockdown, the levels of phosphor-ERK and nuclear-localized NF-κB p65 were reduced in response to JEV infection compared with the levels in cells treated with control siRNA (Fig. 7e,f). Since ERK and NF-κB regulate miR-15b expression via affecting the binding of c-Rel and CREB to the miR-15b promoter, the effect of RIG-I on the binding of c-Rel and CREB to the miR-15b promoter was further explored. ChIP assays were performed in HeLa cells transfected with RIG-I siRNA. The result revealed that c-Rel and CREB binding to the binding sites in the miR-15b promoter were decreased in JEV-infected HeLa cells with reduced RIG-I. In contrast, siNC treatment had no significant effect on c-Rel and CREB binding to miR-15b promoter (Fig. 7g). These findings indicate that the involvement of RIG-I in the upregulation of miR-15b by JEV may through ERK and NF-κB signaling pathway.

## Discussion

MiR-15b is widely expressed and plays diverse roles in different tissues and cell types. It has been found to be highly expressed in tumor cells and be one of the crucial regulators of cell proliferation and apoptosis by targeting cell cycle proteins and Bcl-2 protein, suggesting the potential importance of miR-15b in cancers^40^. miR-15b has also been implicated as important regulators in viral infection through their capacity to regulate both host and viral genes. For example, miR-15b is overexpressed in human papilloma virus (HPV) infected Ca Ski cells, and the elevated miR-15b is regulated indirectly by E2F^41^. Wu *et al*. reported that miR-15b is repressed by hepatitis B virus (HBV) X protein in HepG2 cells and regulates cell growth by targeting FUT2 protein^42^. In addition, miR-15b promotes HBV replication by direct targeting HNF1α in hepatocytes, whereas HBV replication represses the expression of miR-15b^43^. We previously showed that miR-15b is upregulated in JEV-infected glial cells and mouse model. miR-15b appears to have potent pro-inflammatory properties as it enhance RIG-I signaling via targeting RNF125 protein^29^. In the present study, we also found that miR-15b expression was increased in a time- and dose-dependent manner, and miR-15b acted as a positive regulator of the production of viral-induced inflammatory cytokines in HeLa cells with JEV infection. However, little is known about the mechanisms by which miR-15b was regulated in JEV infection.

Similar to protein-coding genes, miRNA genes themselves are subject to sophisticated control. This regulation can occur at several steps: transcription of the primary transcript, pri-miRNA processing by Drosha, export of pre-miRNA, precursor processing by Dicer, or stability of the mature miRNA^44^. The mechanism involved in the activation of miR-15b mediated by JEV infection was investigated in this study. Our results showed that similar trend of mature miR-15b upon increase of its primary transcripts, pri-miR-15b and pre-miR-15b, suggesting that the regulation of miR-15b involves transcriptional mechanisms. Interestingly, we found that the fold changes for miR-15b were higher than the fold changes for the pri-miR-15b and pre-miR-15b following JEV infection in the same time point. This inconsistence may be due to the differential posttranscriptional regulation of miR-15b. Indeed, recent studies have high-lighted the importance of posttranscriptional mechanisms in the regulation of miRNA expression in various cell types^45^. Thus, although our data suggests that JEV-induced miR-15b was regulated transcriptionally, there remains a possibility that expression of miR-15b might also be regulated at the processing level.

The majority of miRNA genes are initially transcribed as primary transcripts by polymerase II in the nucleus and can be elaborately regulated by transcriptional factors^11^. Binding of transcription factors to the promoter elements of miRNAs has been associated with transcriptional regulation of miRNAs in response to microbe infection^13^,^46^. In this study, the minimal promoter region of miR-15b gene was investigated. The data showed that the longest (TSS-2518) and shortest (TSS-559) reporter displayed both basal promoter activity and clear JEV inducibility, but the other three reporters with middle lengths failed to drive JEV-induced promoter activity. One possibility is that the region from −1984 to −559 may contain unknown transcriptional repressor(s) suppressing JEV-induced luciferase expression, and the region from −2518 to −1984 may contain transcriptional activator(s) which counteract with repressor(s) to enhance JEV-induced luciferase expression. However, the molecular mechanism underlying remains to be elucidated in further studies. In addition, functional assays with the miR-15b promoter demonstrated that transcription factors, c-Rel and CREB, are at least partially responsible for transcription of miR-15b in HeLa cells following JEV infection. The results with ChIP assays provided direct evidence that c-Rel and CREB bound to the miR-15b promoter region. Hence, it is possible that transcription factors-mediated transcriptional processing of miR-15b may contribute to the upregulation by JEV. In mammals, c-Rel, RelA (p65), RelB, p50 and p52 are five subunit members of NF-κB family. Promoter binding of c-Rel, p65 and RelB is usually associated with gene regulation^47^. C-Rel has also been involved in transcriptional regulation of some miRNAs^48^. C-Rel along with p65 activates the miR-21 gene promoter and increases miR-21 expression in pancreatic β cells^49^. CREB is a proto-oncogenic transcription factor that promotes tumorigenesis in cancers. As a transcriptional regulator, CREB generally enhances the expression of target genes^50^. Studies showed that some noncoding targets of CREB have been identified. CREB can directly bind to the regulatory sequences of miR-23a and enhance the expression of miR-23a^15^. CREB increases miR-373 expression through the regulation of its promoter^51^. Therefore, transcription factors, c-Rel and CREB, are important in the regulation of miRNAs activation and transcription. In addition to c-Rel and CREB, other transcription factors may also be involved in the transcriptional regulation of miR-15b gene. Specifically, miR-15b has been found to be a direct transcriptional target of E2F, and miR-15b as well as precursor pri-miRNA is elevated upon activation of ectopic E2F1^21^,^32^. In addition, upregulation of miR-15b in HPV infection was regulated indirectly by E2F^41^. How these transcription factors are coordinated to regulate miR-15b gene under physiologic and pathological conditions needs to be further elucidated.

RIG-I, a cytoplasmic sensor for viral RNA, participates in JEV recognition and plays important role in JEV-induced pathogenesis^36^,^37^. Sensing of JEV infection via RIG-I leads to activation of the ERK and NF-κB pathways^36^,^37^. Furthermore, ERK is one potential upstream regulator in transduction of signals to CREB and NF-κB^52^, and CREB is also found to be activated in JEV infection^53^. In addition, c-Rel is a subunit member of NF-κB, and the translocation of c-Rel from the cytoplasm to the nucleus was increased during NF-κB activation^47^. CREB activation and c-Rel nuclear translocation were enhanced following JEV infection. Therefore, we investigated roles of ERK and NF-κB played in regulation of miR-15b expression following JEV infection. Our findings showed that significantly lower levels of miR-15b expression in cells treated with ERK or NF-κB specific inhibitors. Moreover, the binding of c-Rel and CREB to the miR-15b promoter were suppressed by ERK or NF-κB specific inhibitors. The data suggested that ERK and NF-κB pathways are involved in miR-15b induction by JEV infection, presumably through activate transcription factors, c-Rel and CREB, leading to transcriptional activation of miR-15b gene. Furthermore, our data demonstrated that transfection of JEV subgenomic replicon increased the level of miR-15b, whereas JEV proteins and the inactivated JEV infection did not alter miR-15b expression. Since c-Rel and CREB are important transcription factors in regulating the transcription of miR-15b, we speculated that JEV RNA might affect the levels of c-Rel and CREB expression, but JEV protein might not alter the expression level of c-Rel and CREB. However the molecular mechanism underlying remains to be elucidated in further study. It has been reported that several miRNAs are induced by RIG-I. For example, vesicular stomatitis virus (VSV) infection induces miR-146a expression in mouse macrophage in a TRL/MyD88-independent and RIG-I/NF-κB-dependent manner, and miR-155 expression is upregulated in response to RIG-I activation^39^,^54^. In line with these observations, our data also demonstrate that miR-15b induction is dependent on RIG-I since induction of miR-15b by JEV challenge was impaired and binding of c-Rel and CREB to the miR-15b promoter were decreased in RIG-I-knockdown cells. Although no direct interaction of RIG-1 with miR-15b expression machinery was investigated, we speculated that RIG-1 positively regulated the host miR-15b expression may through activation of ERK and NF-κB pathways, leading to the binding of c-Rel and CREB to miR-15b promoter and activating miR-15b transcription. Interestingly, our previous study found that JEV-induced miR-15b can promote RIG-I signaling by targeting a negative regulon of RIG-I, RNF125, leading to upregulation of inflammatory cytokine and type I interferon expression^29^. Thus, we present a new virus-host interaction model that JEV infection is initially sensed by RIG-I, and subsequently activates ERK and NF-κB pathways, which increase the transcription of miR-15b gene. The RIG-I-dependent miR-15b enhances RIG-I signaling as a positive feedback regulator in JEV infection. Our data broaden and deepen the understanding of the roles of miR-15b in the interaction between host and JEV.

In summary, our results show that miR-15b is induced in HeLa cells after infection with JEV, and the upregulation of miR-15b is transcriptionally regulated. These findings also suggest that c-Rel and CREB bind to the miR-15b promoter and modulate miR-15b transcription. Finally, we found that JEV infection upregulates miR-15b expression through RIG-I-dependent manner, and ERK and NF-κB pathways are involved in miR-15b induction.

## Methods

### Reagents

Pharmacological inhibitors of MEK (PD98059), ERK (U0126), p38 MAPK (SB203580), and JNK (SP600125) were obtained from Calbiochem-Merck. NF-κB inhibitor (MG132) was obtained from Sigma. All of the inhibitors were dissolved in dimethylsulfoxide (DMSO, Sigma). Antibodies against RIG-I, TLR3, phosphor-ERK, ERK, NF-κB p65, Lamin A, GAPDH and β-tubulin were purchased from Abclonal technology (Wuhan, China). HRP-conjugated anti-mouse/rabbit secondary antibodies (Boster, Wuhan, China) were used.

### Virus preparation

The JEV wild-type strain P3 used in this study was propagated in suckling mouse brain. The titer of virus was determined by plaque assay on Baby Hamster Syrian Kindey (BHK-21) cells as described previously^55^. To inactivate JEV, virus suspension was exposed to a short wavelength ultraviolet radiation for 2 hours on ice. Stock virus was stored at −80 °C until use.

### Constructs and Plasmids

The miR-15b promoter reporter plasmids TSS-2518, TSS-1984 TSS-1472, TSS-1024, and TSS-559, which contains the corresponding nucleotides proximal promoter sequences of miR-15b, respectively, were constructed by PCR amplification using genomic DNA of human cervical carcinoma cells (HeLa cells) as templates and subsequently cloned into PGL3-basic (Promega, Madison, WI). To construct transcription factors (TFs, including c-Rel, CREB) expression vector, the coding regions were amplified from cDNA derived from HeLa cells and cloned into pCDNA3, to yield pCDNA-TFs. The miR-15b promoter constructs containing site-specific mutation or deletion for transcription factor binding site were constructed by overlap-expression PCR^56^. The plasmid carrying JEV subgenomic replicon was described previously^57^. Primers used for PCR in this study are listed in Table 1. All constructs were verified by sequencing.

### Cell culture and treatment

HeLa cells were obtained from ATCC and were cultured and maintained in Dulbecco’s Modified Eagle’s Medium (DMEM) supplemented with 10% heat-inactivated fetal bovine serum (FBS) and 100 U/ml penicillin, 100 μg/ml streptomycin sulfate at 37 °C in 5% CO_2_. A total of 2 × 10^4^ HeLa cells were seeded onto each well of 48-well plates and incubated overnight, and then transfected with promoter plasmids using lipofectamine 2000. After 24 h, the cell medium was removed and infected with medium, JEV (1 MOI) for the indicated times. Cells were treated with various pharmacological inhibitors (10 μM) before JEV infection, when necessary.

### RNA interference

Small interfering RNA (siRNA) against RIG-I and TLR3 and a non-silencing control sequence without homology to known mammalian genes were purchased from Genepharma (Shanghai, China). The sequence for RIG-I siRNA was: 5 ′-GAGGUGCAGUAUAUUCAGG-3 ′^58^. The sequence for TLR3 siRNA was: 5 ′-CGAAUUUGACUGAACUCCA-3 ′^59^. Non-specific control siRNA sequence, 5 ′-UCCUCCGAACGUGUCACGU-3 ′. Transfection was performed with lipofectamine 2000. Cells were transfected with 50 nM of each siRNA. Cellular RNA and proteins were collected to assess knockdown efficacy.

### RNA extraction and quantitative real-time PCR

Total RNA was extracted from treated cells with TRIzol (Invitrogen) according to the manufacture’s instructions. For detection of pri-miR-15b, pre-miR-15b, pre-miR-16-2, SMC4, TLR3 and RIG-I, 1 μg of RNA was used to synthesize cDNA using a first-strand cDNA synthesis kit (TOYOBO, Osaka, Japan) with gene specific primers for pri-miR-15b, pre-miR-15b and pre-miR-16-2, and the oligo dT primer for β-actin, SMC4, TLR3 and RIG-I. Quantitative real-time PCR (qRT-PCR) analysis was performed using a 7500 Real-time PCR System (Applied biosystems) and SYBR Green PCR master mix (TOYOBO, Osaka, Japan). Amplification consisted of 2 min at 50 °C and 5 min at 95 °C, followed by 40 cycles of 95 °C for 15 s, 60 °C for 15 s and 72 °C for 30 s. Data were normalized according to the level of β-actin expression in each sample using the 2^−ΔΔCt^ method^60^. The detection of miR-15b and miR-16-2 were performed as described previously^29^. Primers for qRT-PCR were listed in Table 2.

### Promoter Analysis

HeLa cells were co-transfected with 100 ng of full-length or a series of truncated or mutant promoter firefly luciferase reporter constructs and 10 ng of renilla luciferase vector (pRL-TK). Luciferase activities were determined with the Dual-Luciferase Reporter Assay System (Promega) according to the manufacturer’s protocol and expressed as relative luciferase activity by normalizing firefly luciferase activity against Renilla luciferase activity.

### Western blotting

Total cellular lysates were generated by lysing cells in radioimmunoprecipitation assay (RIPA) buffer containing protease and phosphatase inhibitors (Roche). Protein concentrations were measured by a BCA Protein Assay Kit (Thermo Scientific). Sodium dodecyl sulphate-polyacrylamide gel electrophoresis (SDS-PAGE) was performed, followed by protein transfer to polyvinylidene fluoride membranes using Mini Trans-Blot Cell (Bio-Rad). Blots were probed with relevant antibodies and detection of proteins was conducted using ECL reagent (Thermo Scientific).

### Chromatin immunoprecipitation assay (ChIP)

ChIP assay was performed according to the manufacture’s protocols of ChromaFlash High-Sensitivity ChIP kit (epigentek, Farmingdale, NY) with minor modifications. A total of 2 × 10^6^ HeLa cells were transfected with HA-c-Rel or HA-CREB expression vector or siRNA for 24 h, or treated with specific inhibitors, and then the cells were then infected with or without JEV. Treated cells were harvested at 24 h post infection by crosslinking in a final concentration of 1% formaldehyde. Crosslinking was stopped after 5 min by adding glycine to a final concentration of 125 mM. The cells were then sonicated to a fragment size range of 100–700 bp. Immunoprecipitation was performed by incubating sheared chromatin overnight at 4˚C with HA tag antibody (Abcam; rabbit origin), c-Rel antibody (Cell Signaling Technology; rabbit origin), CREB antibody (Abcam; rabbit origin) or rabbit antiserum (immunoglobulin G control). The ChIP DNA was extracted and one tenth of the purified sample was subjected to PCR amplification with primer pairs spanning transcription factors binding sites (Table 2). PCR products were resolved by 2% agarose-ethidium bromide gel electrophoresis and visualized by UV. The expression level of a target DNA sequence was determined relative to its abundance in the input chromatin and represented as fold enrichment compared with uninfected control.

### Statistical analysis

All experiments were repeated at least three times with similar results. Analyses were conducted using Graphpad Prism (5.0) (GraphPad Software, San Diego, CA). Results were expressed as mean ± SD. Data were compared either by 2-way ANOVA, with subsequent *t* tests using a Bonferroni post-tests for multiple comparisons, or by a Student *t* test when appropriate. For all tests, p < 0.05 was considered significant.

## Additional Information

**How to cite this article**: Zhu, B. *et al*. Transcriptional regulation of miR-15b by c-Rel and CREB in Japanese encephalitis virus infection. *Sci. Rep*. **6**, 22581; doi: 10.1038/srep22581 (2016).

## Supplementary Material

Supplementary Information

## Figures and Tables

**Table 1:** Primers used for PCR.

Primer Name	Application	Sequence 5′-3′
TSS-2518 F	PCR	CCCTCGAGACTCTGAACCATCACAATCT
TSS-1984 F		CCCTCGAGCATCTTTCCACTACATTGTGC
TSS-1472 F		CCCTCGAGCGATATTGAGTCTACGCTTATG
TSS-1024 F		CCCTCGAGCAATCACCTACCACCAGAT
TSS-559 F		CCCTCGAGATCCAGTGATGGCTACAAC
TSS R		TGGCGCCTAAAATACAAACTC
D1 F	PCR	CCCTCGAGCCAGAGGTGAAGGGATTTAC
D2 F		CCCTCGAGTCAAGTTCCAGGAAAGCG
D3 F		CCCTCGAGGTGACTTCTAAGAGTTACGC
D4 F		CCCTCGAGCTCTTCTGAAGAGGCGTTT
D R		TGGCGCCTAAAATACAAACTC
Mut 1 F	PCR	CAACTTCCAAACCCTCTTCAATCCAGGTCCAGAGGTGAAGG
Mut 1 R		CCTTCACCTCTGGACCTGGATTGAAGAGGGTTTGGAAGTTG
Mut 2 F		CAGGTCCAGAGGTGAAAACATTTAGGTCAAGTTCCAGGAAAGCGGTG
Mut 2 R		CACCGCTTTCCTGGAACTTGACCTAAATGTTTTCACCTCTGGACCTG
Mut 3 F		CAAATACCTGGTACCTCCCGCAGTCCATAGTGACTTCTAAGAGTTACG
Mut 3 R		CGTAACTCTTAGAAGTCACTATGGACTGCGGGAGGTACCAGGTATTTG
Mut 4 F		CGATACTTCTACGCGGCAGCAGTTCCGAGTGGGCCCGAGACCCCG
Mut 4 R		CGGGGTCTCGGGCCCACTCGGAACTGCTGCCGCGTAGAAGTATCG
c-Rel CDS F	PCR	CGGGATCCATGGCCTCCGGTGCGTATAACCCGTATATA
c-Rel CDS R		CCGCTCGAGTACTTGAAAAAATTCATATGGAAAGGAG
CREB CDS F		CGGGATCCATGACCATGGAATCTGGAGCC
CREB CDS R		CGGAATTCATCTGATTTGTGGCAGTAAAGG

**Table 2:** Primers used for ChIP and qRT-PCR.

Primer Name	Application	Sequence 5′-3′
miR-15b promoter (ChIP) F	ChIP	AACCCTTTTCTCACCAGGTCCA
miR-15b promoter (ChIP) R		TTCAGAAGAGGGCGGGCTC
pri-miR-15b F	qRT-PCR	CTTCTGTCTATCACATAAGTGG
pri-miR-15b R		GGTCCAAGTCAATTCCATG
pre-miR-15b F		GGCCTTAAAGTACTGTAGC
pre-miR-15b R		CCTTAAATTTCTAGAGCAGC
pre-miR-16-2 F		CTTGTTCCACTCTAGCAGCAC
pre-miR-16-2 R		GTCACACTAAAGCAGCACAGTA
miR-16-2 F		TAGCAGCACGTAAATATTGGCG
miR-16-2 R		GTGCAGGGTCCGAGGT
SMC4 F		ACGGCATAGTGAACGAGAAA
SMC4 R		GTCAGGGGCTGTAGCAAGTA
RIG-I F		TTCCCACAAGGACAAAAG
RIG-I R		GCCTGTAACTCTATACCCAT
TLR3 F		TCCCAAGCCTTCAACGACTG
TLR3 R		GGGTTTGCGTGTTTCCAGAG
β-actin F		AGCGGGAAATCGTGCGTGAC
β-actin R		GGAAGGAAGGCTGGAAGAGTG
