# Supplementary material for: Transcriptional regulation of miR-15b by c-Rel and CREB in Japanese encephalitis virus infection
Source: Sci Rep. 2016 Mar 2;6:22581. doi: 10.1038/srep22581 (PMC4773857; doi:10.1038/srep22581)
Supplement: Supplementary Information [file srep22581-s1.pdf]

Supplementary information

Title: Transcriptional regulation of miR-15b by c-Rel and CREB in Japanese encephalitis virus infection

Bibo Zhu, Jing Ye, Usama Ashraf, Yunchuan Li, Huanchun Chen, Yunfeng Song & Shengbo Cao*

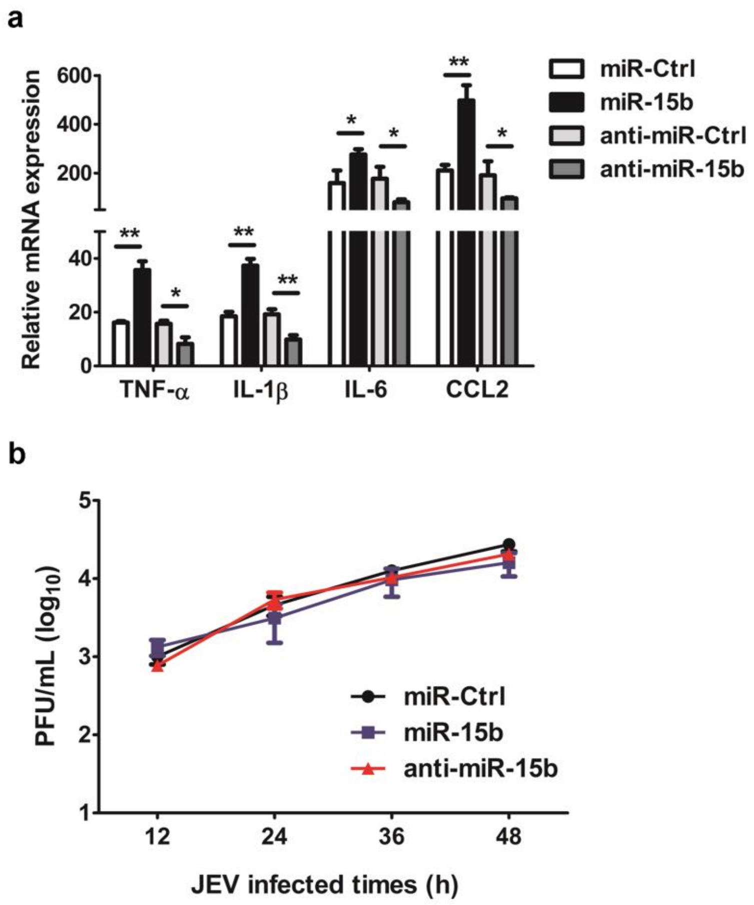

Supplemental Figure 1. The effects of miR-15b upon JEV infection. (a) HeLa cells were transfected with mimics control (miR-Ctrl), miR-15b mimics (miR-15b), inhibitors control (anti-miR-Ctrl), or inhibitors (anti-miR-15b) for 24h and then infected with JEV at an MOI of 1 for 36h. The levels of TNF-α, IL-1β, IL-6, and CCL2 were analyzed using qRT-PCR. (b) HeLa cells were transfected as in (a) and infected with JEV at an MOI of 1 for the indicated times. The titers of infectious virus in the culture supernatants were detected by plaque assay. Error bars represent the standard deviation (SD) calculated from results of at least three independent experiments. Statistical analysis was carried out by 2-way ANOVA with subsequent *t* tests using a Bonferroni post-tests.

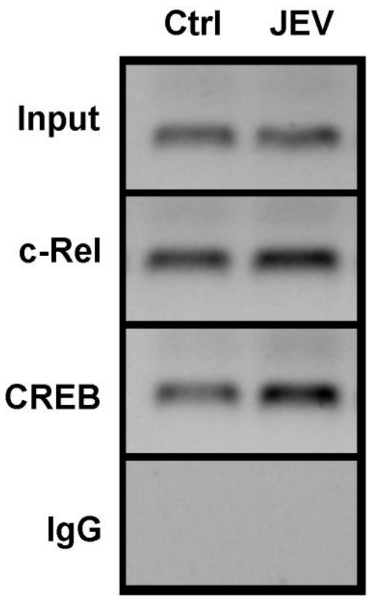

Supplemental Figure 2. JEV infection enhanced the binding of c-Rel and CREB to the miR-15b promoter. HeLa cells were infected with or without JEV for 24h. ChIP assays were performed on cell extracts using antibodies against c-Rel, CREB or rabbit IgG, followed by PCR amplification of the region containing both c-Rel and CREB binding site in the miR-15b promoter. PCR products were separated by acrylamide gel electrophoresis.

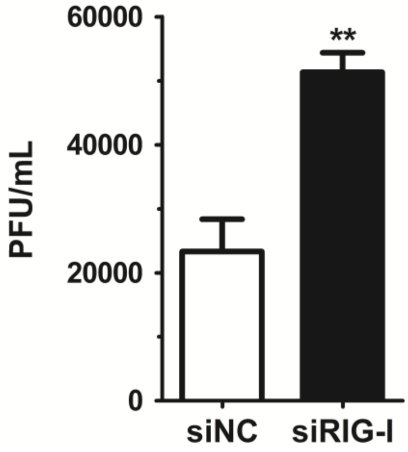

Supplemental Figure 3. Viral load in RIG-I knockdown cells. HeLa cells were transfected with control siRNA or RIG-I siRNA for 24h, and then infected with JEV at an MOI of 1 for 48h. The titers of infectious virus in the culture supernatants were detected by plaque assay. Error bars represent the standard deviation (SD) calculated from results of at least three independent experiments. Statistical analysis was carried out by a Student t test. **, $p<0.01$.
